# Inpatient Delirium in Guillain-Barré Syndrome: Frequency and Clinical Characteristics in a Mexican Hospital

**DOI:** 10.7759/cureus.19260

**Published:** 2021-11-04

**Authors:** Juan Carlos López-Hernández, Maria E Briseño-Godinez, Esther Y Pérez-Valdez, Raul N May-Mas, Javier A Galnares-Olalde, Victoria Martínez-Angeles, Jesus Ramírez-Bermudez, Elizabeth León-Manriquez, Gerardo Chavira-Hernández, Edwin Steven Vargas-Cañas

**Affiliations:** 1 Neuromuscular Diseases, Instituto Nacional de Neurología y Neurocirugía Manuel Velasco Suárez, Mexico City, MEX; 2 Neurology, Instituto Nacional de Neurologia y Neurocirugia Manuel Velasco Suárez, Mexico City, MEX; 3 Neurology, Instituto Nacional de Neurología y Neurocirugía Manuel Velasco Suárez, Mexico City, MEX; 4 Neuropsychiatry, Instituto Nacional de Neurología y Neurocirugía Manuel Velasco Suárez, Mexico City, MEX

**Keywords:** guillian-barré syndrome (gbs), psychosis, advanced age, outcome, neuropsychiatric, delirium

## Abstract

Background

Delirium has a prevalence of 14%-56% in hospitalized patients. Risk factors include advanced age, invasive mechanical ventilation (IMV), and prolonged intensive care unit stay. Neuropsychiatric symptoms have been reported to be related to autoimmune responses secondary to Guillain-Barré syndrome (GBS) with direct involvement of the central nervous system (CNS) or to delirium. There are few reports of the frequency of delirium in patients with Guillain-Barré syndrome (GBS).

Objective

To describe the frequency of and the characteristics associated with delirium in patients with GBS.

Material and methods

A single-center, cross-sectional study was conducted in patients with GBS diagnosis between 2015 and 2019. The diagnosis of delirium was made using the Diagnostic and Statistical Manual of Mental Disorders, 5th Edition (DSM-5) criteria. We compared patients with and without delirium. We performed both univariate and multivariate analyses to identify factors associated with delirium.

Results

A total of 154 patients with GBS were included, of which 20 (12.9%) fulfilled the DSM-5 criteria for delirium. The mean age was 48 ± 18.2 years, the median Medical Research Council (MRC) sum score was 29.3 ± 21.9 points, 65% had bulbar cranial nerve involvement, 80% presented autonomic dysfunction, 85% had ICU stay, and 90% had mechanical ventilation requirement. In the multivariate analysis, the following were the independent factors for the development of delirium: age ≥ 60 (odds ratio (OR): 5.7; 95% confidence interval (CI): 1.3-23.5), time from symptom onset to admission ≤ 3 days (OR: 4.3; 95% CI: 1.1-16.8), autonomic dysfunction (OR: 13.1; 95% CI: 3-56), and intensive care unit stay (OR: 9.5; 95% CI: 2.1-42.6).

Conclusion

Delirium is not frequent in patients with Guillain-Barré syndrome. Patients with advanced age, rapid motor progression, bulbar cranial nerve involvement, prolonged intensive care unit stay, and mechanical ventilation need are more likely to present delirium.

## Introduction

Guillain-Barré syndrome (GBS) is the most common cause of acute flaccid paralysis worldwide, with an incidence of one to four cases per 100,000 person-years. The incidence is higher in men than in women and increases with age. Furthermore, 30% of patients with GBS have severe clinical forms, including rapid progression of weakness, bulbar muscle involvement, and respiratory insufficiency, which need intensive care unit (ICU) monitoring [1,2].

Delirium occurs in 14%-56% of hospitalized patients with any medical condition, increasing hospital stay and mortality. The most common risk factors for delirium are advanced age and preexisting mild cognitive impairment/dementia [3]. Other predisposing factors include male sex, poor vision and hearing, depressive symptoms, and laboratory abnormalities. On the other hand, precipitating factors include drugs such as sedatives or anticholinergics, severe pain, infections, invasive mechanical ventilation (IMV), prolonged ICU stay, and surgery [4].

Neuropsychiatric symptoms, such as psychosis, sleep disturbances, and agitation, are common in patients with GBS. The pathophysiological origin of these symptoms is currently controversial. Some authors report that they are triggered by autoimmune responses that affect directly the central nervous system (CNS), while other authors relate them to delirium [5,6]. The objective of this study is to describe the frequency of and the associated risk factors for delirium in patients with GBS.

## Materials and methods

Design

A single-center, cross-sectional study was conducted in patients with GBS diagnosed between 2015 and 2019 in a neurological reference hospital in Mexico City. GBS diagnosis was made according to the Asbury criteria and delirium according to the Diagnostic and Statistical Manual of Mental Disorders, 5th Edition (DSM-5) criteria [7,8]. Patients who are >18 years old were included. Patients with progression greater than 28 days, intracranial hypertension, central nervous system infection, or epilepsy were excluded.

Measures

A systematic register of clinical characteristics was made, including age, gender, time since symptom onset to diagnosis, history of previous infection, cranial nerve involvement, Medical Research Council (MRC) sum score at the time of diagnosis, ICU admission and length of stay, requirement and days of IMV, and autonomic dysfunction. Prolonged mechanical ventilation was defined as ventilation for more than 30 days. Cerebrospinal fluid (CSF) reports were obtained, and elevated protein concentration was defined as >45 mg/dL. Electrophysiologic subtypes were defined using the Rajabally criteria [9].

Statistical analysis

For descriptive analysis, variables were described as mean, standard deviation (SD), or median and interquartile range according to distribution. Categorical variables were described as frequencies and percentages. To assess group differences, x^2^ and Fisher's tests were used for categorical variables, and Student's t-test or Mann-Whitney U for continuous variables. A p value < 0.05 was considered statistically significant. Univariate and multivariate analyses were performed to identify risk factors for delirium using binary logistic regression, according to the TRIPOD consensus [10]. The univariate analysis included the following variables: mean age, age ≥ 60 years, time from symptom onset to admission, cranial nerve involvement, bulbar nerve involvement, autonomic dysfunction, ICU stay, and IMV requirement. The variables for binary logistic regression were selected on the basis of the results of the univariate analysis, the researcher’s experience, and literature descriptions. We included the following: age ≥ 60 years, time from symptom onset to admission, autonomic dysfunction, and ICU stay. We assessed the goodness of fit using the Hosmer-Lemeshow test and the model performance using the area under the curve (AUC) analysis. Statistical significance was evaluated at the 0.05 level. The results are reported as odds ratio (OR) with 95% confidence interval (95% CI). All statistical analyzes were performed using SPSS 22.

## Results

A total of 154 patients diagnosed with GBS were included, of which 20 patients (12.9%) fulfilled the DSM-5 criteria for delirium during the hospital stay. Delirium mean presentation was on the fifth (3-21) day. Of the patients, 65% had hyperactive delirium and 35% had hypoactive delirium. The mean age was 48 ± 18.2 years, and 50% were ≥60 years. Of the patients, 65% were admitted for ≤3 days since symptom onset. The median MRC score at admission was 29.3 ± 21.9 points, bulbar nerve involvement was present in 65% of the patients, and 80% presented autonomic dysfunction. Furthermore, 85% of patients were admitted to the ICU, and 90% required IMV. The most common electrophysiological subtype reported was acute motor axonal neuropathy (AMAN) (45%). Twenty patients fulfilled the classic Miller-Fisher criteria, although only eight had anti-GQ1b evaluation. Six patients (75%) had positive GQ1b antibodies. Two patients were diagnosed with Bickerstaff brainstem encephalitis, and 28.5% presented albuminocytological dissociation. Baseline population characteristics are depicted in Table 1.

**Table 1:** Baseline characteristics of patients with GBS Yr: years; no.: number; MRC: Medical Research Council; ICU: intensive care unit; IMV: invasive mechanical ventilation; AIDP: acute inflammatory demyelinating polyneuropathy; AMAN: acute motor axonal neuropathy; ASMAN: acute sensory-motor axonal neuropathy

Characteristics	N = 154
Age – yr	48 ± 18.2
Age > 60 – no. (%)	37 (24)
Male gender – no. (%)	101 (65.5)
Time from symptom onset to admission ≤ 3 days – no. (%)	41 (27)
MRC sum score at diagnosis – points	30.2
Cranial nerve involvement – no. (%)	84 (54.5)
Facial nerves – no. (%)	66 (43)
Bulbar nerves – no. (%)	41 (27)
Cardiovascular dysautonomia – no. (%)	37 (24)
ICU stay – no. (%)	42 (27)
IMV requirement – no. (%)	45 (29)
Prolonged IMV – no. (%)	18 (12)
Pneumonia – no. (%)	32 (21)
Delirium – no. (%)	20 (12.9)
Albuminocytological dissociation – no. (%)	56 (36.3)
Electrophysiological variants:	
AIDP – no. (%)	46/100 (35.4)
AMAN – no. (%)	52/100 (40)
ASMAN – no. (%)	6/100 (4.6)
Inexitable – no. (%)	6/100 (4.6)
Equivocal – no. (%)	10/100 (7.7)
Time of hospitalization – days	56 (36.3)
Independent gait at three-month follow-up – no. (%)	75/117 (64.1)

When comparing patients who presented delirium and those who did not, significant differences for delirium presentation were identified in age ≥ 60 years, time from symptom onset to admission ≤ 3 days, involvement of bulbar cranial nerves, autonomic dysfunction, ICU stay, and IMV. There were no significant differences between clinical and electrophysiological variants.

Several statistically significant factors in the univariate model were identified (Table 2). In the multivariate analysis, the independent variables associated with delirium were as follows: age ≥ 60 years (OR: 5.7; 95% CI: 1.3-23.5), time from symptom onset to admission ≤ 3 days (OR: 4.3; 95% CI: 1.1-16.8), autonomic dysfunction (OR: 13.1; 95% CI: 3-56), and ICU admission (OR: 9.5; 95% CI: 2.1-42.6). The performance of the multivariate model through the AUC was 0.95 (95% CI: 0.91-0.98; p ≤ 0.001). We identified autonomic dysfunction as an independent risk factor for delirium, including high/low blood pressure, heart rate, and urinary sphincter involvement.

**Table 2:** Characteristics associated with delirium in GBS Yr: years; no.: number; MRC: Medical Research Council; ICU: intensive care unit; IMV: invasive mechanical ventilation; CSF: cerebrospinal fluid

	Univariate analysis				Multivariate analysis	
Characteristics	Delirium (N = 20)	No delirium (N = 134)	p value	OR (95% CI)	OR (95% CI)	p value
Age – yr	56 ± 18.2	44.9 ± 16.4	0.009	1.0 (1.01–1.07)		
Age > 60 – no. (%)	10 (50)	27 (20.1)	0.008	4.1 (1.5–11.2)	5.7 (1.3–23.5)	0.01
Time from symptom onset to admission ≤ 3 days – no. (%)	13 (65)	28 (20.8)	<0.001	6.8 (2.4–18.7)	4.3 (1.1–16.8)	0.03
MRC score at diagnosis – points	29.3 ± 21.9	34.6 ± 16.3	0.19			
Facial cranial nerve involvement – no. (%)	11 (55)	55 (41)	0.33			
Bulbar cranial nerve involvement – no. (%)	13 (65)	28 (20.8)	<0.001	7.0 (2.5–19.2)		
Cardiovascular autonomic dysfunction – no (%)	16 (80)	21 (15.6)	<0.001	21.5 (6.5–70.7)	13.1 (3–56)	0.001
ICU admission – no. (%)	17 (85)	25 (18.6)	<0.001	24 (6.7–90.8)	9.5 (2.1–42.6)	0.003
IMV requirement - no. (%)	18 (90)	27 (20.1)	<0.001	35.6 (7.7–163.1)		
Prolonged IMV – no. (%)	10 (50)	8 (5.9)	0.03			
Increased CSF protein concentration – no. (%)	4/14 (28.5)	52/102 (50.9)	0.15			

## Discussion

Neuropsychiatric symptoms are frequent in the acute setting and during hospitalization in patients with GBS, with anxiety being the most common symptom (82%). Anxiety is generally associated with an adaptive response after functional loss and the need for invasive mechanical ventilation [11,12]. Other neuropsychiatric symptoms include psychosis (hallucinations and delusions) and sleep disturbances, which have a controversial pathophysiological mechanism. Some authors suggest that these symptoms are associated with an autoimmune response that directly affects the central nervous system (CNS), systemic inflammatory response, and stress as an adaptive response and a common risk factor of delirium (infections and laboratory disturbances) [5].

The reported prevalence of delirium is 16.9% in neurological hospitalization services and 14.9% in neurological emergency services, with CNS infections and stroke being the most common causes [13,14]. Delirium was present in 12.9% of our patients.

Patients with GBS who present delirium have the same risk factors as inpatients with delirium, compared to known predisposing and precipitating risk factors [15]. One of the risk factors for delirium is advanced age, as 50% of patients with GBS aged >60 years presented delirium. This is an important variable to consider because GBS has a higher incidence in patients aged ≥50 years, and up to 1.85 cases per 100,000 population are between the age of 60 and 69 years [1-3].

Although GBS is considered classically a peripheral nervous system disorder, clinical variants such as Bickerstaff encephalitis, Miller-Fisher syndrome (MFS), and pharyngeal-cervical-brachial variant are associated with central involvement such as somnolence [16]. Interestingly, patients with Bickerstaff encephalitis present altered awake status due to the involvement of the ascending reticular system. When these patients are treated (IVIG or plasma exchange) by improving their awake state, they present hyperactive delirium, which does not improve with antipsychotics (haloperidol, quetiapine, and risperidone). In our population, two patients with MFS/Bickerstaff encephalitis presented hyperactive delirium, which was managed with dexmedetomidine with good response.

Some authors suggest that, in patients with GBS, hyperproteinorrhachia associated with mental status abnormalities is due to CNS inflammatory features. Nevertheless, in GBS, 90% of patients present elevated CSF proteins at the third week of symptom onset, which represents dorsal root inflammation [5]. In our study, patients with delirium did not present a significant increase in CSF proteins compared with those without delirium. Most patients in our center have a lumbar puncture performed within the first week of symptom onset. This might explain the low percentage of patients with hyperproteinorrhachia.

In our multivariate model analysis, we identified autonomic dysfunction as an independent risk factor for delirium, including high/low blood pressure, heart rate, and urinary sphincter involvement. Autonomic dysfunction is more common in patients with GBS who present psychosis and REM sleep disturbances. It is secondary to myelinated nerve root inflammation in the thoracic and lumbar autonomic ganglion chains [2]. Autonomic dysfunction usually leads to ICU admission with potential pharmacological intervention. In our population of patients with GBS who presented delirium, 80% presented cardiovascular autonomic dysfunction.

We observed that early admission (≤3 days) from symptom onset is an independent risk factor for delirium. This clinical finding can be explained by the following reasons. First, patients admitted for ≤7 days have faster motor progression and usually have a more severe presentation. Early admission is a risk factor for IMV, as demonstrated by the Erasmus GBS Respiratory Insufficiency Score (EGRIS) scale, where ≤3 days from symptom onset and admission is a variable that predicts respiratory insufficiency [17]. Second, patients who require IMV and have bulbar cranial nerve involvement frequently have aspiration pneumonia in up to 80% of cases. Both IMV and pneumonia are also independent risk factors for delirium.

Limitations of this study include the small number of patients and the lack of clinimetric measures of delirium beyond the use of the DSM-5 criteria.

## Conclusions

It is important to consider delirium in patients with Guillain-Barré syndrome. Patients with advanced age (>60 years), rapid progression of weakness, bulbar cranial nerve involvement, ICU admission, and IMV needs are more likely to present delirium. Prevention and early detection are important to reduce mortality and hospital stay in these patients.
